# A hitchhikers guide to the Galápagos: co-phylogeography of Galápagos mockingbirds and their parasites

**DOI:** 10.1186/1471-2148-11-284

**Published:** 2011-10-03

**Authors:** Jan Štefka, Paquita EA Hoeck, Lukas F Keller, Vincent S Smith

**Affiliations:** 1 Entomology Department, Natural History Museum, Cromwell Road, SW7 5BD, London, UK; 2 Faculty of Science, University of South Bohemia and Biology Centre ASCR, Institute of Parasitology, Branisovska 31, 37005 Ceske Budejovice, Czech Republic; 3 Institute of Evolutionary Biology and Environmental Studies, University of Zurich, Winterthurerstrasse 190, 8057 Zurich, Switzerland

## Abstract

**Background:**

Parasites are evolutionary hitchhikers whose phylogenies often track the evolutionary history of their hosts. Incongruence in the evolutionary history of closely associated lineages can be explained through a variety of possible events including host switching and host independent speciation. However, in recently diverged lineages stochastic population processes, such as retention of ancestral polymorphism or secondary contact, can also explain discordant genealogies, even in fully co-speciating taxa. The relatively simple biogeographic arrangement of the Galápagos archipelago, compared with mainland biomes, provides a framework to identify stochastic and evolutionary informative components of genealogic data in these recently diverged organisms.

**Results:**

Mitochondrial DNA sequences were obtained for four species of Galápagos mockingbirds and three sympatric species of ectoparasites - two louse and one mite species. These data were complemented with nuclear EF1α sequences in selected samples of parasites and with information from microsatellite loci in the mockingbirds. Mitochondrial sequence data revealed differences in population genetic diversity between all taxa and varying degrees of topological congruence between host and parasite lineages. A very low level of genetic variability and lack of congruence was found in one of the louse parasites, which was excluded from subsequent joint analysis of mitochondrial data. The reconciled multi-species tree obtained from the analysis is congruent with both the nuclear data and the geological history of the islands.

**Conclusions:**

The gene genealogies of Galápagos mockingbirds and two of their ectoparasites show strong phylogeographic correlations, with instances of incongruence mostly explained by ancestral genetic polymorphism. A third parasite genealogy shows low levels of genetic diversity and little evidence of co-phylogeny with their hosts. These differences can mostly be explained by variation in life-history characteristics, primarily host specificity and dispersal capabilities. We show that pooling genetic data from organisms living in close ecological association reveals a more accurate phylogeographic history for these taxa. Our results have implications for the conservation and taxonomy of Galápagos mockingbirds and their parasites.

## Background

Parasites represent evolutionary hitchhikers on their hosts with evolutionary histories of each lineage often running in parallel [e.g. [1,2]]. When the hosts speciate, those parasites which are host specific may also become reproductively isolated, potentially leading to co-speciation. Analysis of these host-parasite associations is analogous to reconstructing the evolution of genes tracking organisms, and organisms tracking geological and geographical changes [3,4]. Parasites can also serve as an independent source of information when evolutionary data on the host is insufficient [5]. Host specific ectoparasites have proven to be good proxies for resolving host population structure [6]. This is particularly true when an ectoparasite's life-cycle is entirely bound to a host individual resulting in co-speciation [e.g. [7-10]]. Nevertheless, co-speciation cannot be assumed in all systems where there are high levels of host specificity [e.g. [11,12]] and careful investigation is required to explain the complex associations between hosts and parasites.

A theoretical framework relating co-phylogenetic patterns with population genetic processes was defined by Rannala and Michalakis [4], who formulated assumptions under which congruence between host and parasite genealogies might be expected. These include the assumption of 1) no migration events between splitting populations and 2) coalescence of both host and parasite lineages in the ancestral population. This is difficult to achieve in situations where historical host-parasite associations may have been affected by recurrent migrations or climatic oscillations [e.g. [13,14]], which is common in the fauna of mainland biomes.

Due to their relative isolation from mainland biota, restricted surface area and low probability of multiple colonization events, oceanic islands like Galápagos provide a convenient model to study co-phylogenetic patterns in hosts and parasites. Founding populations of island colonists are typically small in size and can only carry a limited number of gene alleles leading to rapid coalescence. Consequently the effects of selection and genetic drift quickly lead to genetic differentiation and the formation of new species [15].

The Galápagos islands, and Galápagos mockingbirds (*Mimus spp*.) in particular, have played a prominent role in research on island speciation. It was Charles Darwin's observation on the distinctiveness of Galápagos mockingbirds from their mainland relatives that provided much of the founding evidence for the idea that species evolve through time [16].

**Figure 1:**
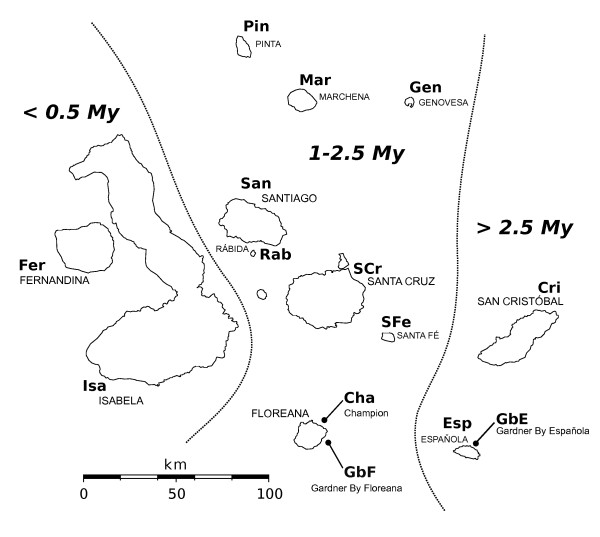
**Map of the Galápagos islands**. Approximate geological age of the archipelago is provided (My = million years) based on literature data [18]. The islands are divided into three zones bordered by a dashed line. Genovesa island is sometimes estimated to be more recent than shown here [e.g. [89]]. Abbreviations of the island names are consistent with those used across all figures.

The geological origin of the Galápagos is very well understood [17-19]. This permits a detailed investigation of the impact of geographic isolation on the formation of population structure and speciation in Galápagos biota. Similar to other archipelagos that arose in the form of a successive chain of volcanic islands (e.g. Hawai'i), it has been proposed that the pattern of speciation by endemics follow the successional origin of islands in the chain, a phenomenon sometimes called the progression rule [20]. Geological evidence shows a strong northwest to southeast gradient in the age of the Galápagos islands (Figure 1). The youngest rise above a volcanic mantle hotspot in the northwest of the archipelago, and as they migrate along a tectonic plate transition towards the Southeast, the islands age and shrink due to erosion [18]. Thus the youngest islands (Isabela and Fernandina) are less than 0.5 million years (My) old, whereas the oldest extant islands (San Cristóbal and Española) are more than 2.5 My. Submerged islands of up to 14 My are found east of Española [17,21] suggesting a much earlier origin is possible for at least some Galápagos biota. However, only a few instances of endemic fauna older than the age of the extant islands have been confirmed, such as the *Galapaganus *weevils [22]. A vast majority of terrestrial species are younger than 2.5 My and also fit the progression rule pattern of colonization [for a review see [23]].

Exceptions to the progression rule include species capable of long distance dispersal, such as Darwin's finches [24] and some species of winged insects [25,26], both of which show complex colonization histories. Arbogast et al. [27] showed that the mitochondrial DNA (mtDNA) phylogeny of mockingbirds largely follows the progression rule. This corresponds with low levels of long-distance dispersal as detected by population genetic surveys [28]. Comparing the level of divergence in Galápagos mockingbirds with mutation rates commonly found for coding mtDNA genes in birds Arbogast et al. [27] estimated that their colonisation of the Galápagos falls well within the age of present islands, and the distribution of the clades amongst particular islands was generally congruent with patterns of island age.

**Figure 2:**
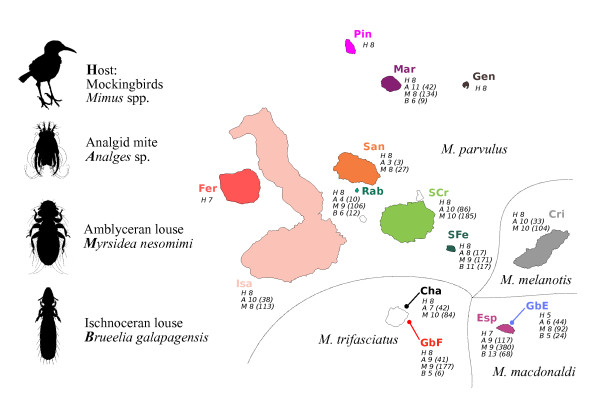
**Geographic distribution of sampled hosts and parasites**. Numbers of sequenced specimens for the host (denoted with *H*) and each parasite taxon (denoted using first letter of the respective generic name) are listed under each island name. Total number of parasite specimens collected on each island are in parentheses. The island colour scheme used here is consistent with those used across all subsequent figures. Mockingbird allopatric species distribution is indicated by the fine dashed lines.

Arbogast et al. [27] also showed that the mtDNA data only partially fit the traditional taxonomy of Galápagos mockingbirds. Morphologically there are four nominal species of Galapágos mockingbirds, each with distinct geographic distributions. The Hood mockingbird (*Mimus macdonaldi*) inhabits Española, the San Cristóbal mockingbird (*M. melanotis*) is found on the island of the same name and the Floreana mockingbird *M. trifasciatus *is present on two islets adjacent to Floreana. The rest of the archipelago is populated by the Galápagos mockingbird (*M. parvulus*) (Figure 2). Despite belonging to three different nominal species, birds from the eastern islands of the archipelago (Española, San Cristóbal, and Genovesa) possess similar mtDNA haplotypes, while populations from Isabela in the west of the archipelago, are genetically more divergent from other *M. parvulus *populations than previously expected [27]. A study conducted by Hoeck et al. [28] shows that nuclear genetic data obtained using microsatellites largely agrees with the morphological distinction of species. Thus, at least some of the mtDNA differences are an exclusive feature of the mtDNA genealogy while other mtDNA sequences agree with the traditional taxonomy.

Discrepancies between mitochondrial and nuclear genealogies have been observed on multiple occasions in phylogeographic studies during the past two decades [29]. Several processes can explain these divergences, with secondary contact of previously separated lineages, ancestral allelic polymorphism and horizontal gene transfer representing the most common reasons for discordance. Typically, the age of the mtDNA lineages or alleles of nuclear genes predate the time of the population separation [30]. Therefore, the genetic pool of colonists may contain several copies of these haplotypes or alleles during the colonization event. Over time, random genetic drift leads to fixation of different mtDNA haplotypes and nuclear alleles in different populations. This ultimately leads to discordant genealogies amongst different loci, which hamper the interpretation of phylogeographic data based on analysis of single genes [31].

In this paper we reconstruct the common phylogeographic patterns shared between mockingbirds and their parasites inhabiting Galápagos islands using mtDNA and nuclear data. We evaluate the level of ancestral polymorphism affecting species phylogenies based on mtDNA data. Our analyses are partitioned by populations of all four mockingbird species and three associated parasites: an Astigmatid mite (*Analges *sp.) which has yet to be taxonomically described, an amblyceran parasitic louse (*Myrsidea nesomimi*) and an ischnoceran parasitic louse (*Brueelia galapagensis*). All three species of parasites are Galápagos endemics and are commonly associated with Galápagos mockingbirds [[32,33] authors' observation]. Despite sharing the same host spectrum, the three parasites represent three phylogenetically unrelated lineages [34,35] that differ in their ecology. Feather mites of the genus *Analges *feed predominantly on feathers and are strictly host specific [36]. The two genera of chewing lice (*Myrsidea *and *Brueelia*) are known to differ in their feeding habits and in their levels of host specificity, which is strict in *Myrsidea *and more relaxed in *Brueelia *[e.g. [32,37]]. This assemblage of parasitic taxa allows us to discern shared evolutionary signal from unique evolutionary events affected by varying life history traits.

## Methods

### Material collection

Collection of both the host and parasite material was perfomed between 2004 and 2008 in the frame of previous research [28]. Mockingbird blood samples were obtained from 14 Galápagos islands and the birds were examined for ectoparasites on 11 of the 14 islands (see Figure 2). *Myrsidea *and *Analges *samples were found on all 11 islands, whereas *Brueelia *was found only on 6 islands. Birds were captured using mist nets or potter traps and a small blood sample was collected from a small puncture on the wing vein. Ectoparasites were collected from the plumage of the birds using dust ruffling [38]. Birds were ringed to prevent resampling and released immediately after sample collection. A single parasite specimen was analysed for each parasite taxon per host individual, with seven exceptions (four for *Brueelia*, two for *Analges *and one for *Myrsidea*) when the host infection rate was exceptionally low. For a complete list of analysed samples including GPS coordinates of the sampling localities and GenBank accession numbers see table in Additional file 1.

DNA extraction of parasite samples was performed using Qiagen's MicroDNA extraction kit following the voucher method of Cruickshank et al. [39]. After DNA extraction voucher specimens were either mounted on permanent slides or preserved in ethanol. MtDNA sequences of mockingbirds were amplified from DNA extractions made from blood samples previously used for a microsatellite population genetic study [28].

### Mitochondrial and nuclear DNA sequencing

We sequenced a homologous 1050 bp fragment of the Cytochrome oxidase subunit I (COI) gene in the mockingbirds and all three parasite taxa using a combination of previously described universal primers and dedicated primers based on sequences of related taxa available from GenBank (see Table 1 for primer sequences). The PCRs contained 20 μl volume of: 1 to 2 μl of extracted DNA solution, 5 pM of each primer, 15 mM MgCl_2_, 10 mM concentration of each dNTP, and 0.25 units of Taq polymerase. The PCR profile was as follows: 5 min at 95°C followed by 35 cycles of 1 min at 94°C, 45 sec at the annealing temperature specified below and 1 min 15 sec at 72°C. The final elongation step was performed for 10 min at 72°C. The annealing temperatures were 57°C for *Mimus*, 48°C for *Analges *and *Brueelia*, and 50°C for *Myrsidea*. To obtain complementary information on the population structure of ectoparasites independent of mitochondrial DNA, the Elongation factor 1 alpha (EF1α), a nuclear gene, was sequenced in two parasites, *Brueelia *and *Analges*. EF1-For3 and Cho10 primers [40] were used for both PCR amplification and sequencing. The PCRs contained the same concentrations of reagents as above and the PCR profile was as follows: 5 min at 95°C followed by 30 cycles of 1 min at 94°C, 40 sec at 54°C and 45 sec at 72°C. The final elongation step was performed for 10 min at 72°C. EF1-For3 and Cho10 primers did not reliably amplify in *Myrsidea *and thus the EF1α gene was not analyzed for this species. Prior to sequencing COI and EF1α PCR products were purified using Alkaline Phosphatase and Exonuclease I enzymes according to manufacturer's protocol (New England Biolabs). The COI PCRs were sequenced using newly designed species-specific internal primers (Table 1) aligning from the middle part of the sequence and extending towards the ends. To check for consistency of sequence reads at the ends, approximately 15% of sequences were also sequenced from 5' and 3' ends using PCR primers. Sequencing was performed either on an ABI 3730 sequencer (Applied Biosystems) or using a commercial service (Macrogen Inc., South Korea). Sequence contigs were prepared in Seqman (DNAstar) and alignments were created manually in Seaview 4.2 [41] without need for gap adjustments. Individual sequences were collapsed into haplotypes using Collapse 1.2 [42].

**Table 1:** PCR primers for amplification of the COI gene.

Species	Primer name	Direction	Primer sequence	Reference
*Mimus*	BirdF1_Nes	F	AACCAACCACAAAGATATCGGCAC	Modified from BirdF1 [87]
	COIH_Nes	R	GGGCTACTACGTAGTAAGTGTCATGT	Modified from COI H7005 [74]
	Nes_InF	F	CTCACCGACCGCAACCTCAA	This study
	Nes_InR	R	GGATAGTATGGCTCATACTATTCCTATGTA	This study
*Analges*	An_F	F	ATATCCACTAATCACAAAGATATTG	This study
	COI H7005	R	CCGGATCCACNACRTARTANGTRTCRTG	[74]
	An_InF	F	CCGTAATTTTAATTCTACTTTTTTTG	This study
	An_InR	R	CAAACCCAGGTAAAATCAAAATATA	This study
*Myrsidea*	Myr_F	F	ATATTGGYACTCTYTAYTTAATCTTTGGTT	This study
	COI H7005	R	CCGGATCCACNACRTARTANGTRTCRTG	[74]
	Myr_InF	F	CCGAAATTTTAATACCTCTTTCTTTG	This study
	Myr_InR	R	AGATTATACCAATGACCCCAAAACT	This study
*Brueelia*	COI LCO1490	F	GGTCAACAAATCATAAAGATATTGG	[88]
	COI H7005	R	CCGGATCCACNACRTARTANGTRTCRTG	[74]
	Bru_InF	F	TCGTAATTTGAATTCTTCTTTTTTTG	This study
	Bru_InR	R	ACCCAAAAACTTCCTTCTTTCC	This study

### Amplification of mockingbird microsatellites

400 *Mimus *individuals covering the spectrum of 11 islands available for parasite sampling were genotyped using microsatellites. Extraction of DNA from blood on filter paper and amplification at 26 microsatellite loci (MpAAT18, 25, 26, 45, 83 and 96, and Nes01, 03, 04, 05, 06, 07, 08, 10, 11, 12, 13, 14, 15, 16, 17, 18, 19, 20, 22 and 23) were performed as described previously [43]. Microsatellite loci were amplified in six independent multiplex reactions (Panel A-D, Hoeck et al., [43]; Panel E with MpAAT18, 25, 45 and 83; Panel F with MpAAT26 and 96 under the same PCR conditions as Panel B & C). Fragment analyses were performed on a 3730 DNA Analyser using Gene-Scan-500 LIZ size standard (ABI) and Genemapper v.4 software (ABI) followed by manual proofreading of genotypes.

### Phylogenetic analysis of sequence data

Three approaches were employed for genealogical reconstruction using COI haplotypes in *Mimus*, *Analges *and *Myrsidea*. Due to a very low level of sequence polymorphism *Brueelia *was excluded from the phylogenetic analysis. Neighbor Joining (NJ) trees of haplotypes were obtained in PAUP*4.0b10 [44]. Maximum Likelihood (ML) phylogeny was reconstructed using PhyML 2.4.4 [45], and MrBayes 3.1 [46] was employed to perform Bayesian Inference (BI) from the data. In MrBayes two independent runs with 4 chains each and 30 million MCMC replications were performed for each dataset. The first 3 million replications at the beginning of the runs were discarded as burnin. The model of molecular evolution for all analyses was selected according to AIC and BIC criteria in jModeltest 12.2.0 [47]. HKY+I+G was selected for *Analges*, HKY+G for both *Myrsidea *and *Mimus*. Sequences of *Analges sturninus *(Genbank no.:GQ864342), *Myrsidea eisenrati *(AF545731) plus *Mimus gundlachii *(EF484222) and *M. gilvus *(EF484220), were used respectively as outgroups for the *Analges*, *Myrsidea *and *Mimus *datasets. The COI haplotype data and ML treefiles have been deposited in TreeBASE (http://www.treebase.org; study accession number S11770).

The branch support of NJ and ML phylogenies was obtained with 1000 bootstraps of the data in PAUP and PhyML, respectively. Convergence between estimated values of model parameters obtained in independent BI runs and their effective sampling sizes were checked using Tracer 1.5 [48]. Convergence of inferred BI topologies was inspected using AWTY [49].

Due to allelic variability and frequent occurrence of heterozygotes in EF1α sequences of *Analges*, the allelic phase of specimens could not be unequivocally reconstructed from sequences obtained through direct sequencing. The heterozygous sites in the DNA sequence resulted in ambiguous nucleotides. Hence the EF1α sequences were not analysed phylogenetically. Instead each position in the alignment for each specimen was scored either as homozygous or heterozygous (when ambiguous). Then the distribution of heterozygous or homozygous states for each site within the alignment was compared across island populations. These data were contrasted with gene genealogies obtained from the COI data. A similar approach was adopted for exploring the *Brueelia *EF1α dataset, which was, on the contrary, extremely uniform and contained only one informative mutation (see Results section).

### Genealogy and population genetic statistics

Due to extremely low levels of genetic variability in the COI *Brueelia *sequences, this dataset was not analysed using traditional phylogenetic reconstruction. Instead genealogical information of *Brueelia *and the other taxa was extracted from a haplotype network built using the program TCS 1.2 [50]. Estimates of genetic divergence were performed in Mega 5.0 [51]. For each taxon we calculated overall mean genetic distances (p-distances) [52] and pairwise genetic distances between the islands. The software DNASP 5.1 [53] was used to calculate per island statistics of haplotype diversity and nucleotide variability, and to perform neutrality tests (Tajima's D, Fu and Li's D) for all four organisms. Significance of the values obtained in neutrality tests was tested with 10,000 coalescence simulations of the data.

### Microsatellite data analysis

To obtain an estimate of population structure in the mockingbirds that is independent of mtDNA genealogy, mockingbird microsatellite data were analysed using the Bayesian clustering algorithm in Structure 2.3.3 [54,55]. This analysis complements the estimates of genetic variability and differentiation presented in Hoeck et al [28]. The Bayesian clustering analysis was performed on an extended microsatellite dataset and provides an estimate of population structure by assigning individual specimens to genetic clusters. The analysis was run using 500,000 MCMC replications as a burnin followed by 1 million replications from which posterior distributions were drawn. To test for possibility of intra-island population structure the number of clusters (K) modelled in the analysis was increased by one (to 12), compared to the 11 sampled islands. An admixture ancestry model with the assumption of correlated allele frequencies was used as a prior in the analysis. 15 independent runs of the analysis were performed to check for consistency of the results. Mean values of log likelihood, L(K), and the ΔK statistics of Evanno et al. [56] were used to determine the optimum number of genetic clusters. Graphic visualisation of the results was prepared in Distruct 1.1 [57].

### MtDNA reconstruction of shared evolutionary history

*BEAST [58] is a recently introduced extension of BEAST 1.6 [59] and supports Bayesian analysis allowing simultaneous estimation of phylogeny and node age. *BEAST was employed to perform comparison of mutation rates between *Mimus *and their *Analges *and *Myrsidea *ectoparasites. This software was originally developed to infer species trees from multilocus data, and was used here to reconcile the evolutionary history of the three organisms. This was achieved by inferring a multi-species tree from gene trees obtained from *Mimus*, *Analges *and *Myrsidea*, for which sampling was available on all 11 islands. Because no missing information on any of the analysed species-island combinations is allowed in the analysis, the *Brueelia *dataset containing data on only 6 islands was excluded. Datasets containing COI sequences of all available specimens (i.e. not collapsed into haplotypes) were used for all *BEAST analyses. These were run selecting the same models of molecular evolution as in the phylogenetic analysis. In the first analysis relative evolutionary rates between the three gene trees (*Mimus*, *Analges*, *Myrsidea*) were estimated. A prior of 1.0 was set on the rate for *Mimus*, allowing relative estimation of rates for the parasites. Clock-like behaviour of the *Mimus *dataset was tested in baseml, a program from the PAML package [60] and it was rejected (X^2 ^= 39.44, df = 23, P < = 0.02). Therefore uncorrelated lognormal relaxed clock priors were selected for rate comparisons. The analysis was run for 100 million MCMC replications and a speciation birth-death process was selected as a tree prior. Two independent runs were performed and convergence between the estimates of parameters was assessed using Tracer 1.5 [48]. Results of the two runs were combined in Logcombiner 1.6.1 [59] with 10% burnin. A separate analysis was performed to estimate the topology of the species tree and the dates of the co-speciation events. In this analysis the age of the root of the species tree was defined with a lognormal prior, using a mean set to 2.9 million years ago (Mya) and standard deviation set to 0.4. This calibration prior sets the highest probability of the age of the root to the estimated geologic age of Española, the oldest extant island of the archipelago [18] but also allows for an earlier origin on the submerged islands east of Española [17]. An uncorrelated relaxed clock with uninformative priors was selected for the three gene trees. Two independent runs were conducted with 500 million MCMC replications each. As above the convergence between runs was inspected in Tracer 1.5 [48] and results were combined in Logcombiner 1.6.1 [59]. We are aware that restricting the analysis with only one calibration point is insufficient to provide precise estimates of the node ages. However, our aim was solely to provide ordinal information on the succession of origin of the fauna in particular regions of the archipelago, rather than to provide exact dates of diversification events.

Complementary to the *BEAST analysis, the tree topologies of *Mimus *and its parasites (*Analges *and *Myrsidea*) were reconciled using the program Jane [61]. This program allows mapping parasite trees onto host phylogeny using a heuristic approach with the so-called Genetic Algorithm. The TreeMap costs model [62] was used to score the numbers of evolutionary events (co-speciations, host switches, duplications and losses). Then the statistical significance of the cost of identified co-speciations is tested using permutation analysis. Tree topologies obtained in the ML analysis and pruned to contain only the main mitochondrial lineages were supplied to the program. Mapping was run with Genetic Algorithm set to 500 generations with population size 300. The permutations were run both randomising the tips of the trees and randomising the parasite tree topology using the sample size of 1000.

### Geophylogenies

To visualise the level of congruence between gene genealogies and incorporate information on the geographic distribution of the specimens, mtDNA phylogenies were converted to KML files, which can be viewed using the freely available Google Earth application [63]. The online tool GeoPhylo 2-4 [64] was used to convert ML phylogenies of *Mimus*, *Analges *and *Myrsidea*, and a multi-species tree into KML files. For the visualisation purposes a NJ tree of *Brueelia *was generated in PAUP*4.0b10 [44] using JC distance model and converted into KML format. The root of the tree was assigned according to a mutation split identified in the EF1α sequences that divides the populations into two groups - the Española and the rest of the archipelago (see Results section). Rooting with an outgroup was not possible in *Brueelia *due to the very low genetic diversity of Brueelia samples and a lack of sequences for close relatives in GenBank.

## Results

107 sequences of the COI gene were obtained in *Mimus*, 86 in *Analges*, 98 in *Myrsidea*, and 45 in *Brueelia*. These sequences were collapsed into 25, 71, 37 and 8 haplotypes, respectively. 51 EF1α sequences were obtained in *Analges *and 29 in *Brueelia*. The list of sequenced specimens and associated haplotype numbers are available in Additional file 1.

### Genetic comparison of populations

COI sequence data show that *Analges *is genetically the most diverse organism, both in terms of number of haplotypes (Hd) and nucleotide diversity (Pi), followed by *Myrsidea *(Table 2). *Mimus *shows considerably smaller levels of population variability, while *Brueelia *is the most conserved taxon with only a few mutations spread across the 1050 bp alignment and each island population made up of one to three haplotypes. This is reflected in the values of genetic divergence (p-distances) seen for the four taxa (Additional file 2). The overall mean genetic divergence is highest for *Analges *followed by *Myrsidea*, *Mimus *and *Brueelia*. Similarly a comparison of distances between particular islands shows that the values are in general highest for *Analges *and lowest for *Brueelia*.

**Table 2:** Genetic diversity of sampled populations and neutrality test results.

Island	**No. Specim**.				**No. Haps**.				Hd				Pi				Tajima's D/Fu&Li's D			
	
	*H*	*A*	*M*	*B*	*H*	*A*	*M*	*B*	*H*	*A*	*M*	*B*	*H*	*A*	*M*	*B*	*H*	*A*	*M*	*B*
**Española**	7	8	9	13	4	8	7	2	0.714	1.000	0.893	0.318	0.0011	0.0090	0.0016	0.0014	**-1.43/-1.51**	-0.59/0.02	-1.28/-0.13	-1.32/-0.37
**Gardner by Española**	5	6	8	5	1	5	4	1	0.000	0.933	0.714	0.000	0.0000	0.0087	0.0017	0.0000	x	0.45/0.51	0.61/-0.07	x
**San Cristóbal**	8	10	10	.	2	9	2	.	0.250	0.978	0.467	.	0.0002	0.0074	0.0005	.	-1.05/-1.13	-0.59/-0.67	0.82/0.80	.
**Champion**	8	7	10	.	1	3	1	.	0.000	0.667	0.000	.	0.0000	0.0008	x	.	x	0.21/-0.06	x	.
**Gardner by Floreana**	8	9	9	5	1	6	3	2	0.000	0.833	0.417	0.400	0.0000	0.0025	0.0007	0.0008	x	-0.43/-0.30	**-1.51/-1.68**	-0.97/-0.97
**Santa Fé**	8	8	9	11	1	6	5	2	0.000	0.893	0.417	0.182	0.0000	0.0018	0.0019	0.0002	x	-1.36/-1.36	-0.56/-0.37	-1.13/-1.29
**Santa Cruz**	8	10	10	.	2	10	4	.	0.250	1.000	0.583	.	0.0005	0.0115	0.0010	.	-1.30/-1.41	-0.70/-0.44	-1.15/-0.28	.
**Santiago**	8	3	8	.	3	3	4	.	0.464	x	0.643	.	0.0005	x	0.0007	.	-1.30/-1.41	x	**-1.45/-1.57**	.
**Rábida**	8	4	9	6	6	3	2	1	0.893	0.833	0.500	0.000	0.0060	0.0011	0.0005	0.0000	**1.76/1.53**	0.52/0.59	0.99/0.84	x
**Isabela**	8	10	8	.	2	9	7	.	0.536	0.978	0.964	.	0.0005	0.0101	0.0053	.	1.17/0.89	-0.59/-0.68	0.03/0.29	.
**Marchena**	8	11	8	6	4	9	4	3	0.786	0.945	0.643	0.600	0.0044	0.0038	0.0029	0.0006	-0.10/0.37	**-2.03/-2.39**	-1.05/-0.88	-1.13/-1.16
**Fernandina**	7	.	.	.	2	.	.	.	0.286	.	.	.	0.0003	.	.	.	-1.01/-1.05	.	.	.
**Genovesa**	8	.	.	.	1	.	.	.	0.000	.	.	.	0.0000	.	.	.	x	.	.	.
**Pinta**	8	.	.	.	2	.	.	.	0.250	.	.	.	0.0002	.	.	.	-1.05/-1.13	.	.	.

Abbreviations and comments: "Hd" haplotype diversity; "Pi" nucleotide diversity. Values of significant neutrality tests (P < 0.05) are in bold. "." no sampling for a given taxon; "x" statistics not performed due to lack of variability (< 2 haplotypes) or too few samples (< 4); species abbreviations (*H*, *A*, *M*, *B*) as in Figure 2.

Neutrality tests performed separately for each island population for all taxa generally show moderately negative values, and are only significantly different from expected values in a few cases. Populations showing significantly negative values of both Tajima's D and Fu&Li's D are Española in *Mimus*, Marchena in *Analges *and Santiago and Gardner by Floreana (GbF) in *Myrsidea *(Table 2). Negative values caused by an excess of low frequency polymorphisms usually indicate population size expansion after an earlier bottleneck or a selective sweep [65]. Only the *Mimus *population on Rábida shows significantly positive values (again for both statistics) suggesting population shrinkage or an effect of balancing selection. These tests could only be performed for populations containing two or more haplotypes, thus many *Mimus *populations comprising single haplotypes could not be tested because they lacked variability (Hd and Pi = 0, Table 2). The Champion and Gardner by Española (GbE) population of *Myrsidea*, and the Rábida populations of *Brueelia *similarly comprise single haplotypes.

### Mitochondrial phylogenies

**Figure 3:**
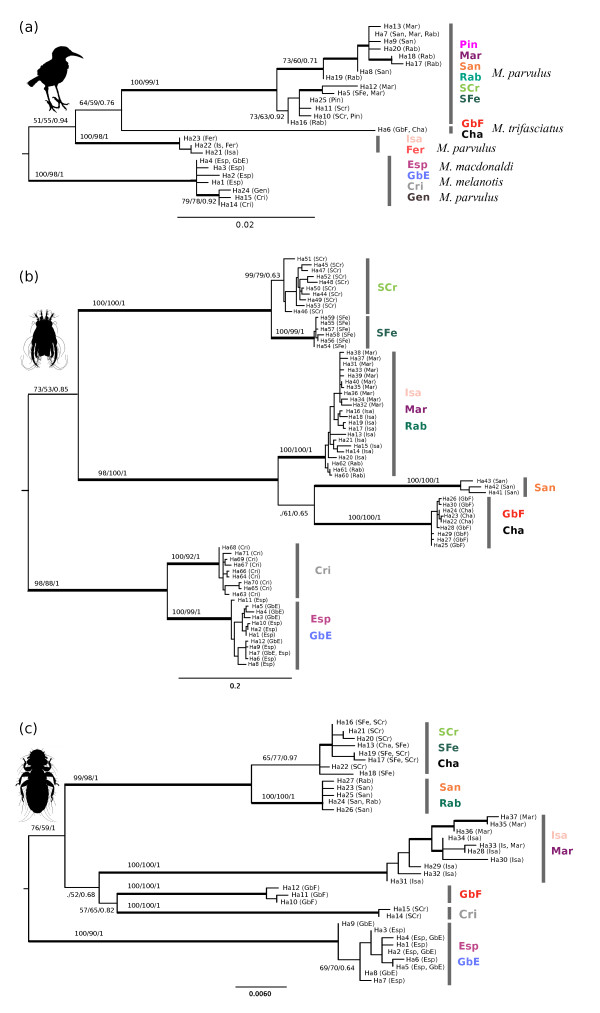
**Maximum likelihood phylogenies of COI haplotypes**. (a) *Mimus*, (b) *Analges *and (c) *Myrsidea*. Bootstrap values and posterior probabilities (NJ/ML/BI) for major clades are indicated. Clades with bootstrap support above 75% and BI posterior probabilities higher than 0.95 are depicted in bold. Outgroups are not shown.

COI topology of the *Mimus *haplotypes (Figure 3) is largely congruent with the earlier results of Arbogast et al [27], who analysed a different set of mockingbird samples using shorter fragments of the COI gene in combination with 2 other mtDNA loci. The most basal split separates mockingbird populations inhabiting the South-East islands (Española, GbE, San Cristóbal and Genovesa) from the rest of the archipelago. Isabela and Fernandina formed another monophyletic lineage followed by GbF and Champion, which comprise a single haplotype. The rest of the islands form a single lineage. This picture largely follows the traditional taxonomy of Galápagos mockingbirds with two exceptions. 1) As in Arbogast et al. [27] the Genovesa population (*M. parvulus*) was grouped into a single clade with the San Cristóbal (*M. melanotis*) and Española (*M. macdonaldi*) populations. 2) Due to the near basal position of Isabela and Fernandina populations, the *M. parvulus *is polyphyletic with respect to *M. trifasciatus*. These data contradict current taxonomic and nuclear DNA (microsatellite) results [28] that group the Genovesa population with the neighbouring *M. parvulus *populations from the North-Western islands of the archipelago. Hoeck et al. [28] also found that the Española and San Cristóbal populations, although closely related, form two clearly distinguishable clusters based on microsatellite distance data.

The topologies of the COI phylogenies obtained for *Myrsidea *and *Analges *sp. parasites broadly follow that of the host phylogeny. The split between populations from the Southeast islands of the archipelago are located at the root in all phylogenies (Figure 3). Most islands or groups of islands form very well supported monophyletic lineages suggesting a deep origin for mitochondrial clades associated with island populations. A few exceptions where the bootstrap and posterior probability support is weaker are also seen. These are usually located near the base of the trees and probably reflect rapid succession of diversification events in ancestral populations of the corresponding mitochondrial lineages. Despite the general congruence of the three phylogenies, the topology of relationships between some of the clades differs both between the two parasites and with their host. Most discordant was the clustering of the Champion population of *Myrsidea *with a clade found on Santa Fé and Santa Cruz. For the other two taxa (*Mimus *and *Analges*), Champion samples were grouped with Gardner by Floreana populations, which was expected, since both islands are small islets adjacent to Floreana and host remnant populations of the endangered Floreana mockingbird that went extinct on Floreana in the late 19^th ^century. This means the two populations are of recent common origin. Indeed, the mockingbird mtDNA sequences show a single haplotype for the two islets, complying with the close relationship and reduced population sizes.

### Population genealogies and EF1α variability

**Figure 4:**
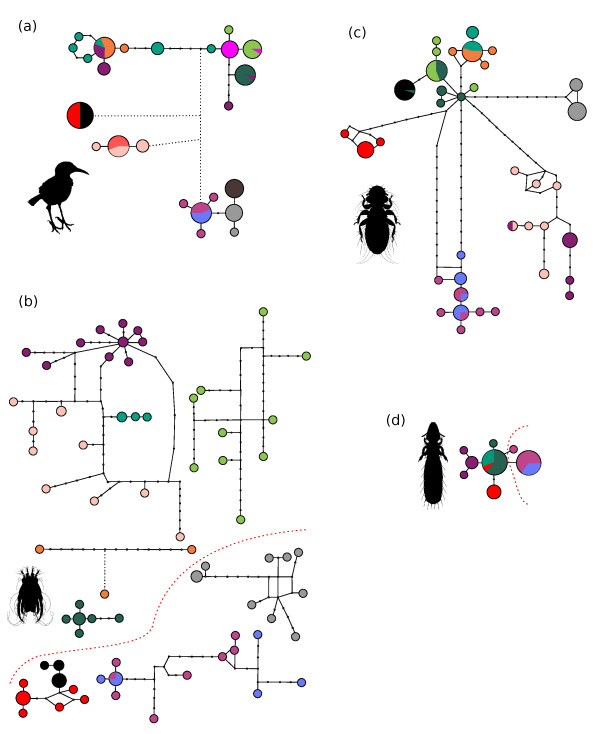
**MtDNA haplotype networks of populations generated with TCS**. (a) *Mimus*, (b) *Analges*, (c) *Myrsidea *and (d) *Brueelia*. Haplotypes are scaled proportionately to the number of samples. Colours of the haplotypes refer to the geographic origin of the specimens (Figure 2). Empty circles mark missing haplotypes along mutational pathways. Dotted lines represent relationships beyond the parsimonious criterion of 14 mutations calculated in TCS. Most island populations in *Analges *are too divergent to be unequivocally linked. Fine dashed red lines in the *Analges *and *Brueelia *networks denote the transition in allelic variability identified from the EF1α sequences.

The pattern of population structure seen in the mtDNA haplotype networks shows several strongly differentiated lineages in *Mimus*, *Myrsidea *and *Analges *(Figure 4). In contrast, the haplotype network of *Brueelia *shows very little sequence variation (Figure 4d). Despite this, several *Brueelia *populations contain exclusive haplotypes (e.g. Española together with GbE and Marchena), highlighting a degree of genetic isolation between the islands. The *Brueelia *dataset of 29 EF1α sequences supports this view. The EF1α gene is almost invariable, out of the 347 bp sequenced, there were only 10 mutated sites, which were mostly singletons. Only one mutation, which was shared among multiple specimens, is informative and it differentiates Española and GbE populations from the rest. This confirms that the deepest genetic separation in *Brueelia *lies between the SE islands and the rest of the archipelago (fine red line in Figure 4b and 4d), as identified in the other three taxa using outgroup sequences (Figure 3).

In contrast to *Brueelia*, the pattern of haplotype distribution in *Analges *shows an extreme level of diversification, with each island (with the exception of GbE) comprising an exclusive set of haplotypes (Figure 4b). Less population structure is seen in *Myrsidea*, where a few haplotypes are shared between Isabela and Marchena, Santa Fé and Santa Cruz, Santiago and Rábida, and one haplotype was shared between Champion and Santa Fé (Figure 4c). One haplotype was also shared between Champion and Santa Fé. Much lower levels of differentiation are found in *Mimus*, where several haplotypes are shared between two or three islands in the central and North-West part of the archipelago. *Analges *also shows extremely high levels of allelic variability in EF1α sequences. The distribution of genetic variation is geography dependent, showing clear structure between the South-East and North-West (Figure 4b). Samples from Española, GbE, San Cristóbal, Champion and GbF only contain a few heterozygous positions in the alignment, with 47 out of the total of 58 mutated sites present as fixed homozygotes. In contrast, populations from the other six islands show a much higher proportion of heterozygous sites (data not shown).

### Microsatellite analysis of mockingbirds

**Figure 5:**
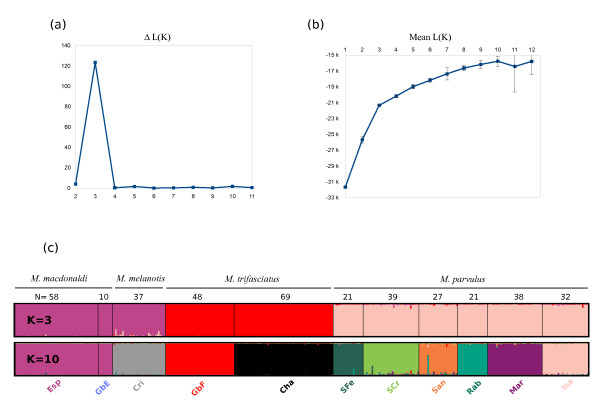
**Microsatellite structure of mockingbird populations**. (a) Evanno et al's ΔK statistics and (b) mean values of L(K) plus/minus standard deviation obtained from 15 runs of the analysis. (c) Results of assignment tests for numbers of clusters K = 3 and K = 10. Individual specimens are represented by vertical bars. Number of samples analysed per each island (N) are provided. Each genetic cluster is represented by a different colour and, where possible, these match the colour of the associated island on the map in Figure 2.

The pattern of population structure in *Mimus *obtained using Structure software shows hierarchical structuring of genetic diversity (Figure 5). The steepest change in the ΔK statistics of Evanno et al. [56] was identified for K = 3 (Figure 5a). The three determined clusters are represented by 1) Española with GbE and San Cristóbal, 2) Champion and GbF, and 3) the rest of the archipelago (Figure 5c). Despite *M. melanotis *from San Cristóbal being clustered with *M. macdonaldi*, there is a slight difference in the genetic composition between the two species: The San Cristóbal population possesses partially mixed genotypes (Figure 5c).

The ΔK statistics are known to pick up the deepest level of genetic structure (i.e. evolutionary oldest) for datasets containing hierarchical data [56]. In contrast L(K) identifies much shallower divergences. The values of the mean L(K) levels off at K = 10 (Figure 5b), where a geographically determined pattern of population structure is seen with all but two of the islands comprising exclusive clusters (Figure 5c). The exception is GbE which shares the same genetic cluster with its parent island of Española. This is almost certainly due to the very small geographic distance between GbE and Española (approximately 1 km) that allows for frequent exchange of migrants. With K = 10 each *Mimus *species belongs to a separate cluster (*M. melanotis *and *M. macdonaldi*) or is split into several clusters (*M. parvulus *and *M. trifasciatus; *Figure 5c).

### Estimation of shared evolutionary signal

**Figure 6:**
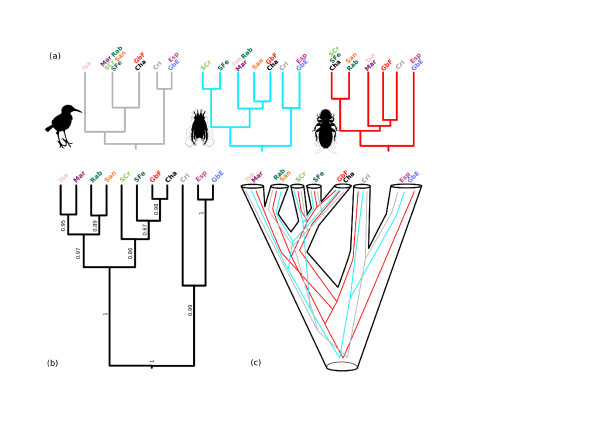
**MtDNA reconstruction of the evolutionary history shared between taxa**. Schematic representation of individual gene trees (phylogenies from Figure 3) are provided (a). The multi-species tree (b) was generated in *BEAST and individual gene genealogies are plotted against putative evolutionary history of taxa based on the topology of the multi-species tree (c).

To reconcile the evolutionary history of the parasites and their hosts, a putative "multi-species" tree was reconstructed in *BEAST. The topology of the resulting tree (Figure 6) is compatible with the geological history of the islands [18], the host microsatellite data in Hoeck et al [28] and the microsatellite analysis presented here. Posterior probabilities [PP] are above 0.95 in all but three nodes (Figure 6b). The branches with lower support are amongst the shortest ones on the tree. One of these branches joins Rabida with Santiago (PP = 0.89), the other two join Santa Cruz with Santa Fe (PP = 0.86) and Santa Fe with the lineage containing Champion and GbF (PP = 0.67). As is the case with the individual mitochondrial genealogies, the basal split lies between the South East (SE) islands (Española, GbE, San Cristóbal) and the rest. The clade containing SE populations is further divided separating Española with GbE from San Cristóbal. A second major clade comprising islands from the central and northern part of the archipelago shows sub-structuring into two lineages. One consists of populations from Champion, GbF, Santa Fé and Santa Cruz, while the other comprises the northern-most islands clustering Isabela together with Marchena and Rábida with Santiago (Figure 6b). Despite several incongruences between individual tree genealogies observed for particular taxa, the independent phylogenetic signals become evident when sequences are jointly analysed. This is best seen when individual genealogies are contrasted with the resulting species tree in Figure 6c, or using a Google Earth visualisation available in Additional File 3. *BEAST was also used to estimate relative evolutionary rates between the host and their parasites. Results are congruent with the differences in genetic variability estimated in DNASP. *Analges *shows the fastest mutation rate and is approximately 9 times faster than *Mimus *(mean = 8.70, 95% Highest Posterior Density interval [HDP]: 6.81-10.00). *Myrsidea *is considerably slower than *Analges *but still significantly faster than its host (mean = 1.87, 95% HDP: 1.09-2.74). Despite the statistically significant result of the baseml test highlighting deviation from clock-like behaviour, the values of standard deviations of the uncorrelated lognormal clock obtained in *BEAST were below 1 for all three taxa (lowest in *Analges *= 0.20, highest in *Myrsidea *= 0.69). Values below 1 indicate only moderate deviation from clock-like behaviour [66], thus our estimates of relative mutation rates were not significantly affected by these deviations.

Estimates of node ages from the multi-species tree have very wide confidence intervals due to the single calibration point (see below). However, observed values lie within the date ranges determined from geological data [18], and are indicative of the time axis for the succession of speciation events. The basal split between the SE islands and the rest of archipelago is within the prior interval, with mean value of 1.20 Mya (95% HDP: 2.22-0.39). Subsequent splits are much younger, with the age of the split between San Cristóbal and Española estimated at 0.43 Mya (95% HDP: 1.01-0.05) and 0.19 Mya (95% HDP: 0.43-0.04) for the split between the two groups of North-Western islands (see Additional file 4 for a complete chronogram). The multi-species estimates probably reflect the final stage in the separation of faunas as opposed to estimates obtained from the same analysis of individual species genealogies that showed slightly earlier separation of mtDNA clades. For instance, the age of the basal split showed values of 1.58 Mya (95% HDP: 3.24-0.41) in *Analges*, 1.54 Mya (95% HDP: 3.14-0.38) in *Mimus *and 1.53 (95% HDP: 3.1-0.39) in the genealogy of *Myrsidea *(Additional file 4).

Results obtained in the reconciliation analysis with Jane show significant level of co-speciation in the *Mimus*-*Analges *association (P < 0.03, in both permutation tests). 4 co-speciations, 2 duplications, 1 host switch and 1 loss were identified. In contrast only 1 co-speciation with 3 host switches and 4 duplications was found for the *Mimus*-*Myrsidea *association. This solution was did not show significant cospeciation in the permutation tests (P < 0.87). More co-speciation events for the *Mimus-Myrsidea *dataset could be manually enforced to reflect better relative timing of splitting diversification events between the two trees. However, these adjustments would come at higher cost because they would inflate the number of losses or host switches. Figures containing mapped phylogenies for both host-parasite associations are available in Additional file 5.

## Discussion

We studied the co-evolutionary patterns between populations of four host and three ectoparasite species living in close ecological association on the Galápagos islands using mtDNA (COI) and nuclear loci (EF1α sequences, microsatellites). Despite varying levels of genetic variability between species and loci, reconstructed phylogeographic patterns show that the population structure between host and parasite lineages is broadly congruent and their diversification is sequentially ordered according to the geological age of each island. In all cases the deepest genetic splits occur between SE islands and the rest of the archipelago (Figures 3 and 6). Most incongruent events between individual gene phylogenies are likely to be attributable to the effect of ancestral polymorphism. Assessing the contribution of stochastic processes on the evolutionary patterns obtained for each species is possible through reconciling the phylogeny of these taxa. Output from this simultaneous analysis agrees with the traditional classification of the mockingbird species and with *Mimus *microsatellite results much more closely than when the *Mimus *mtDNA data are analysed separately.

### Ancestral polymorphism and phylogeography

The occurrence of ancestral polymorphism followed by a process of lineage sorting creates differences in topologies between gene trees and species trees of closely related taxa. When unrecognized, this makes accurate interpretation of genealogical data extremely difficult [e.g. [67]]. Ancestral polymorphism affects both nuclear and mitochondrial genes, but is more problematic for mitochondrial data due to their haploid nature. Despite this, mtDNA remains one of the most valuable resources for phylogeographic inference, mainly due to its fast mutation rate and lack of recombination, which clusters individuals at the intra-specific level [68]. To obtain an unambiguous picture of species history several independent (i.e. nuclear) loci are recommended to be co-analysed with mitochondrial data [29]. Such nuclear loci might include sequences of nuclear genes or multilocus data like AFLP, microsatellites, and SNP's. Unfortunately, nuclear coding genes often do not provide sufficient resolution to detect all relationships in recently diverged taxa, and non-coding fast evolving genes like rDNA spacers (ITS1, ITS2) often create paralogues prone to stochastic evolution [e.g. [69,70]]. Furthermore, developing multilocus markers *de novo *is prohibitively time consuming and expensive when required for multiple taxa.

Here we show that pooling mtDNA data from several organisms, when there is reasonable *a priori *expectation of them having a shared evolutionary history, improves confidence in the inferred phylogeographic patterns. Parasites, and host specific ectoparasites in particular, can predict the population structure of their hosts [e.g [1,71]], and in some cases can reveal more about the host's recent evolution than the host data in isolation. Whiteman et al [72] showed that the population structure of lice parasitizing Galápagos hawks suggests that the hawks (*Buteo galapagoensis*) colonised the archipelago very recently. This is not evident in independent studies of the hawk populations themselves because the genetic differences have not had time to fix. In the case of the mockingbird parasites, the effect of parasite isolation through host specificity is strongly enhanced by physical allopatric isolation of the host populations on different islands. Hoeck et al [28] measured pairwise genetic distances (Nei's Ds) between mockingbird populations from 15 different islands. These data show that populations of mockingbirds occupying separate islands represent distinguishable genetic pools.

A similar picture of strongly isolated mockingbird populations was obtained in the present study using the Bayesian clustering algorithm in the Structure software, where populations are delimited solely by the genetic character of the individuals without prior information on their geographic distribution. This strongly indicates that mockingbirds do not regularly migrate between islands. The ecological dependencies between mockingbirds and their obligate parasites mean that the lice and mites similarly lack opportunities for frequent inter-island migration. Hence, most of the cases where parasite genealogies do not match the inferred multi-species tree (Figure 6) are likely to be a result of ancestral polymorphism in the distribution of haplotypic lineages across the islands, and are not due to recent migrations followed by switches to different host clades. The distribution of genetic diversity in mtDNA haplotypes between the islands strongly supports such a view. For example, nearly all incongruences between particular genealogies are observed in samples from younger North-Western islands where the processes of genetic drift followed by accumulation of mutations has had less time to act. Furthermore, there is evidence that even in the case of younger islands, population structure is not affected by recent migration. For example, *Analges*, which exhibits a remarkably high mutation rate (see discussion below), shows no haplotypes that are shared between any of the islands, including the youngest ones like Isabela, Rábida and Marchena (haplotype network in Figure 4b). Populations of mites on individual islands diverged from each other through mutation and the resulting population structure has not been stirred up by any migration.

There is one possible exception to this general pattern. Despite the close geographic distance and putative recent common origin of Floreana mockingbirds on Champion and GbF [73], the populations of *Myrsidea *on the two islands are strikingly different (Figure 3c). The Champion population constitutes a single haplotype that is very closely related to haplotypes from Santa Fé. The Champion haplotype is even shared with one specimen from Santa Fé (see haplotype network in Figure 4c). This close link suggests that a recent migration event between these islands is a more probable explanation than shared ancestry. Such a migration would require an unknown louse vector since neither Floreana mockingbirds nor *Analges *mites exhibit recent links with Santa Fé. Unfortunately, unlike the other three taxa where microsatellite or EF1α data provide additional clues, no nuclear markers were available for *Myrsidea*, thus resolving this problem with confidence will require additional data. This probably also affected the position of Champion and GbF islands on the multi-species tree (Figure 6b). Nodes joining these islands together with Santa Fe and Santa Cruz have the lowest posterior probabilities on the multi-species tree (0.67 and 0.86 respectively) and this topology is also in conflict with the traditional taxonomy of mockingbirds. The close relationship of the Champion and GbF populations of *M. trifasciatus *with those on Santa Cruz and Santa Fé make the *M. parvulus *populations inhabiting Santa Cruz, Santa Fé and the youngest parts of the archipelago paraphyletic. This contradicts the microsatellite data, which clearly distinguish all *M. parvulus *populations from *M. trifasciatus *on Champion and GbF.

Like other species tree reconciliation methods based on multi-species coalescent models [58], the *BEAST algorithm assumes no horizontal gene transfer in the gene genealogies. In one instance this assumption is violated by our data through the possibility of a recent introduction of *Myrsidea *from Santa Fé to Champion island, which is analogous to a host switch in host-parasite reconciliation methods. This is the only case where the incongruence between the topologies is located on the terminal nodes. We can reasonably suppose that the deeper incongruences are caused by ancient ancestral polymorphism, which is accommodated in the *BEAST analysis. Excluding this terminal event, other instances of incongruence do not significantly affect our interpretation of the reconciled multi-species tree.

An improved fit between the genealogies of *Analges *and *Mimus *compared to the *Myrsidea *- *Mimus *association is also seen in the results produced by the Jane analysis, where only one co-speciation was inferred for the latter pair. Instead Jane identified a relatively large number of host switches or duplications. This highlights a pitfall of the multi-gene *BEAST analysis which cannot accommodate host switching events. Nevertheless, Jane cannot incorporate ancestral polymorphism, which is critical for analysing evolutionary recent associations. For this reason we consider the reconciliation analysis via tree mapping less suitable for the Galápagos dataset. Furthermore, traditional reconciliation analysis is only as good as the supplied trees. By utilising tree topologies instead of raw sequential data for all available specimens, the analysis is limited to the solutions permitted by the tree topologies and cannot assess uncertainty in the input data in a way that is possible in the Bayesian analysis.

### Mockingbird conservation

Understanding the origin of the Champion population of *Myrsidea *is also interesting with respect to the conservation of the endangered Floreana mockingbird. Floreana mockingbirds on Champion and GbF represent remnants of the bird population extinct on Floreana. Bird populations on both islets show dramaticaly low levels of genetic variability, as identified through the haplotype diversity presented here and by Arbogast et al [27]. Microsatellite data generated by Hoeck et al [73] also show very short coalescence times for the populations on the two islets. Nevertheless, both Hoeck et al [28] and the Structure analysis performed here group the two Floreana mockingbird populations together when compared to other Galápagos mockingbird species. More detailed knowledge about the epidemiology and evolutionary history of *Myrsidea *on Champion and GbF would provide valuable data relevant to a tentative reintroduction of mockingbirds on Floreana. Additional genetic loci and extended sampling would help to assess the level of host-parasite co-adaptation in the two populations, and help assess risks connected with uniting the populations on Floreana.

### Louse Taxonomy

Palma and Price [33] identify two subspecies of *Myrsidea nesomimi *within the Galápagos based on morphological data. These subspecies comprise *M. n. nesomimi *(found on Epaňola, GbE and on the two islets of Floreana) and *M. n. borealis *(occupying the rest of the archipelago). Genetic data presented here clearly separate the Epaňola and GbE population of *Myrsidea *from the rest of the archipelago (Figure 3c). However, the Champion and GbF populations seem to be genetically distinct from Española and, at least in the case of GbF, also from the other Galápagos islands.

### Mutation rates

Greater population differentiation has occurred in the parasites (excluding *Brueelia*) than in their hosts (Figure 4). This is congruent with the faster parasite mutation rate identified in the *BEAST analysis. When compared to their hosts, elevated mutation rates are a common feature in lice and might be explained through the shorter louse generation time leading to quicker accumulation of new mutations [74]. Relative evolutionary rates of mockingbird *Myrsidea *are approximately two-fold faster than their hosts, which is in line with the rate difference commonly found in other studies of lice and their vertebrate hosts [e.g. [74-77]]. However, the nine-fold faster mutation rate of *Analges *is unexpectedly high. This remarkable difference requires further attention, not least because the length of the generation time in *Analges *is probably not different to lice. Although exact data on Analgid mites are lacking, the generation times of taxonomically related Sarcoptid mites are two to three weeks [78], similar to most parasitic lice [37]. To our knowledge, there are no studies of feather mites that provided relative mutational rate estimates for comparison. However, the fast genomic evolution in *Analges *is likely to be a lineage specific character. In addition to *Analges*, Astigmatid mites, containing many other parasitic and free-living species, have been shown to have significantly faster mutation rates than other mite groups [35].

### Louse ecology influencing genetic patterns

As representatives of two separate louse suborders the louse species analysed in this study differ considerably with respect to their ecology and evolutionary origins. *Brueelia*, as other ischnoceran lice, feed on feathers, whereas amblyceran *Myrsidea *include host body fluids in their diet. Amblycera are therefore more exposed to the host's immune response than Ischnocera [37]. Such interaction may promote selection towards host specific forms in *Myrsidea *and might accelerate their genetic differentiation relative to ischnoceran *Brueelia*.

Other ecological characters may also explain the different levels of genetic differentiation in these different suborders of parasitic lice. The pattern reported here of lower levels of intra-specific variability in Ischnocera relative to Amblycera has also been identified in other taxonomic studies of lice. For example, Bueter et al. [79] compared levels of intra-generic genetic diversity between *Brueelia *and *Myrsidea *parasitizing thrushes (genus *Catharus*, Passeriformes). Their data show decreased genetic diversity and lack of co-speciation in *Brueelia *when compared to their hosts. This could be explained by increased dispersal capabilities in *Brueelia*, either through direct contact of host organisms or via phoresis (transport) on hippoboscid flies parasitizing various bird hosts [80].

Comparative studies of genetic differences at a very low (intra-specific) evolutionary levels are rare in lice [for exceptions see [81-83]] but in these cases different dispersal capabilities have also been suggested as important factors contributing to the differences in population structure. The role of phoresis in facilitating the dispersal of lice has been documented on numerous occasions in Ischnocera with a majority of cases citing *Brueelia *attached to hippoboscid flies. In contrast, phoretic associations involving Amblycera are very rare [80]. Hippoboscid flies are present on Galápagos mockingbirds [authors' observation] making dispersal through phoresis a possible explanation for the lack of inter-island differentiation in *Brueelia*. However, it is improbable that hippoboscids could migrate between islands without being attached to a bird. Thus any migration of *Brueelia *between islands, whether vectored by hippoboscids or not, is likely to involve another bird host. *Brueelia galapagensis *has been recorded from several other species of hosts including the Small Ground Finch (*Geospiza fuliginosa*) [32,84] which is endemic to the Galápagos and is capable of migration between islands [24,85]. Although records of *B. galapagensis *from non-specific hosts (i.e. other than *Mimus*) have been questioned and may be attributed to contamination [R.L. Palma, personal communication] it is possible that *B. galapagensis *occasionally occurs on the Small Ground Finch as stragglers. The lack of genetic structure in *B. galapagensis *might be explained by phoretic transfer to the Small Ground Finch and the inter-island migration of this species, followed by recurrent phoretic transfer back to mockingbirds.

*Brueelia *also show very little intra-population variability, which is compatible with low population sizes of the parasite recovered during collecting. Despite large numbers of mockingbirds inspected for lice, *Brueelia *was absent on many islands, especially on larger islands of the archipelago and in those instances when *Brueelia *was present, its abundance and prevalence were very low in comparison to *Myrsidea *(Figure 2). This might suggest lower levels of adaption for *Brueelia *on mockingbirds compared to the other two parasitic taxa. We can only speculate as to why *Brueelia *is missing on larger islands. It may be the case that *Brueelia *is only capable of surviving on mockingbirds that exist in smaller, genetically depleted host populations. For instance, a similar link between host genetic diversity and the prevalence of an ischnoceran parasite, was reported in populations of a wild lesser kestrel, *Falco naumanni *and their lice, *Degeeriella rufa *[86].

## Conclusions

Using mitochondrial DNA sequences and nuclear data we studied 400 samples of recently diverged Galápagos mockingbirds and 229 specimens of 3 species of their parasites (*Analges*, *Myrsidea *and *Brueelia*). We found that co-phylogeographic patterns inferred on the level of single gene genealogies (for *Mimus*, *Analges *and *Myrsidea*) are considerably impacted by differential distribution of ancestral polymorphism. In contrast, extremely low genetic variability and lack of co-phylogeographic congruence was found in *Brueelia*. These differences may be explained by life history traits in *Brueelia *such as their dispersal capabilitity, abundance, and lower levels of host specificity. A more accurate picture of the phylogeographic history of these lineages, congruent with the geological history of the islands and with available nuclear data was obtained through a joint analysis of data for the three co-evolving groups. We show that pooling genetic data for several organisms living in close ecological association improves the inference of phylogeographic histories in recently diverged species.

## Authors' contributions

JŠ sequenced the studied taxa, performed computional analyses and drafted the manuscript. VS concieved the idea of studying host-parasite co-evolution in mockingbirds, provided guidance with data analyses and contributed to drafting the manuscript. PH concluded fragment analysis of the mockingbird microsatellites. PH and LK collected all study material and helped with finishing the manuscript text. All authors read and approved the final manuscript.

## Supplementary Material

Additional file 1 **List of samples sequenced for COI and EF1α**. Click here for file

Additional file 2 **Matrices of mean genetic divergence (p-distances) for the COI gene**. Click here for file

Additional file 3 *BEAST chronograms. Provided are multi-species chronogram (a) and chronograms for *Mimus *(b), *Analges *(c) and *Myrsidea *(d) datasets. Tip labels on the multi-species tree are the same as in Figure 1. Tip labels on the individual species trees are abbreviations of the island names from Figure 1 and voucher numbers from Additional file 1. Mean values of ages for major clades are provided with blue bars ranging the 95% highest posterior probability interval. Click here for file

Additional file 4 **Zipped archive containing KML files for each of the taxon phylogenies**. These data files should be viewed in Google Earth [http://www.google.com/earth]. Click here for file

Additional file 5 **Host-parasite phylogenies mapped with Jane software**. Results for *Mimus-Analges *(a) and *Mimus-Myrsidea *(b) associations are shown. Hollow circles mark co-speciations, solid circles mark duplications. Host switches are marked by arrows and losses by dashed lines. Host trees are in black, mapped parasite histories are in blue. Yellow nodes indicate another location of equal cost exists, red nodes mark the solution with the lowest cost. Taxon labels were assigned as species and island abbreviations as shown in Figures 1 and 2. Click here for file
