# Supplementary figures and images for: A hitchhikers guide to the Galápagos: co-phylogeography of Galápagos mockingbirds and their parasites
Source: BMC Evol Biol. 2011 Oct 3;11:284. doi: 10.1186/1471-2148-11-284 (PMC3209464; doi:10.1186/1471-2148-11-284)

(a)

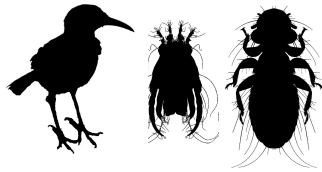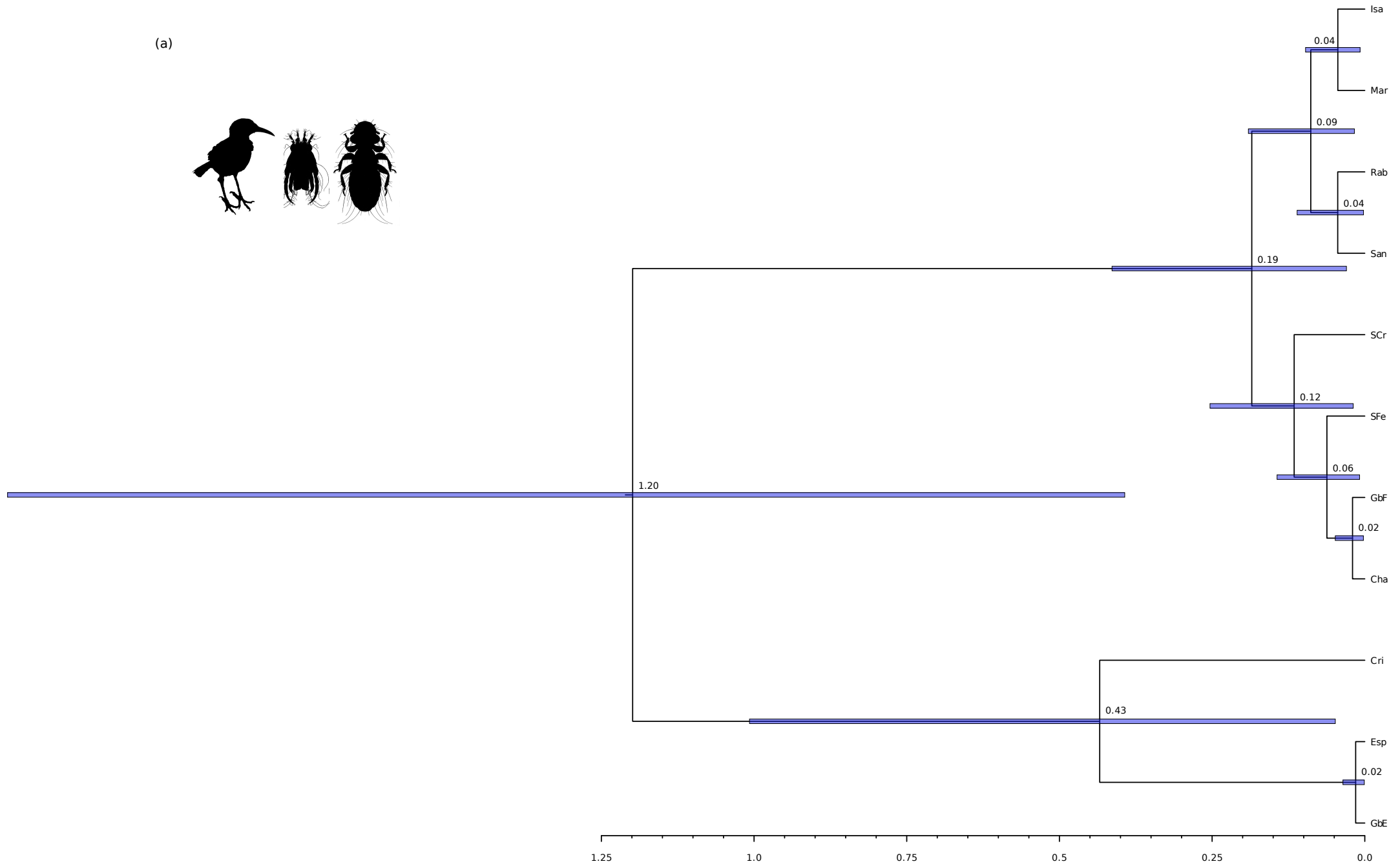

(b)

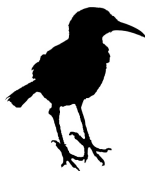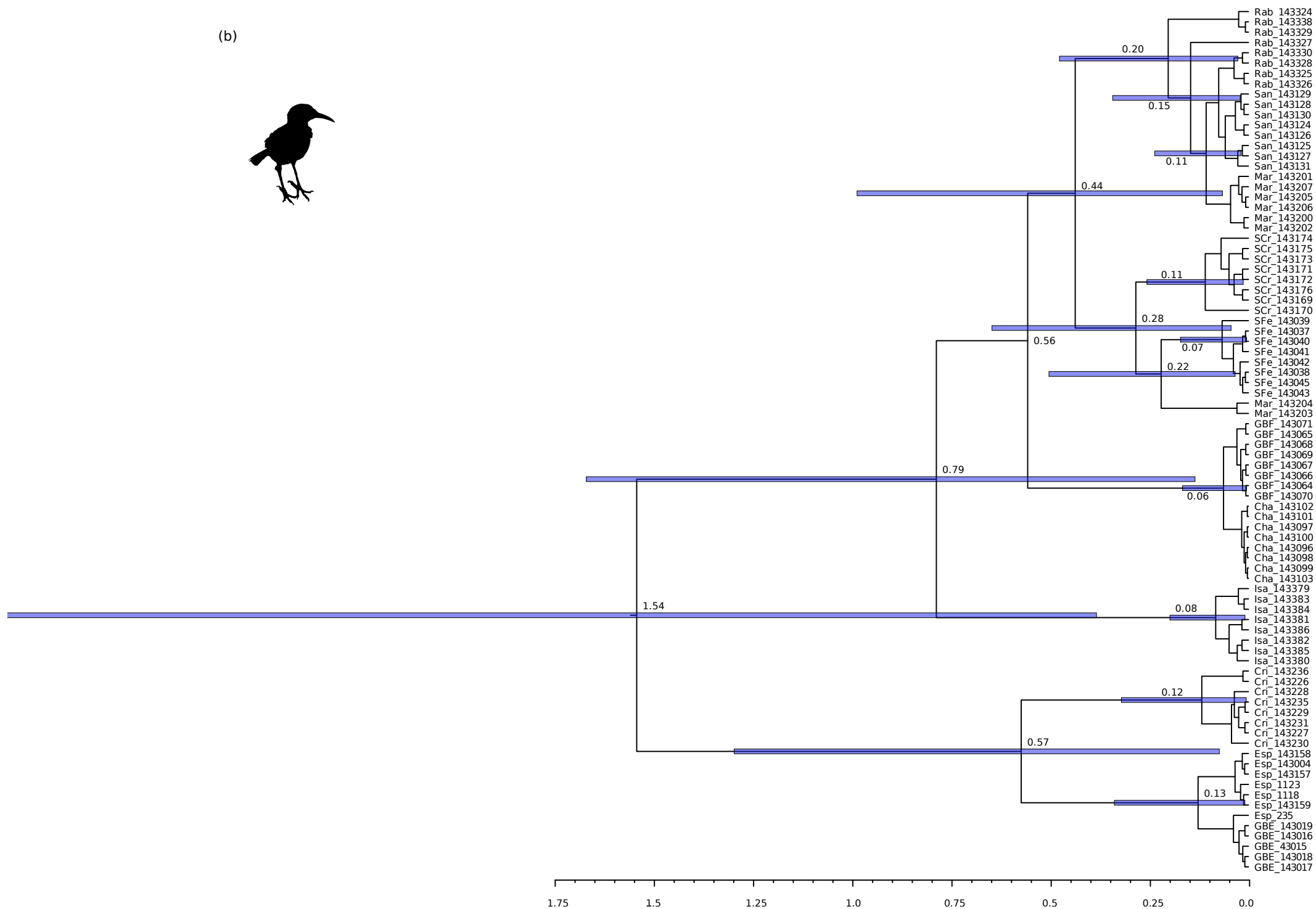

(c)

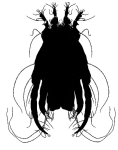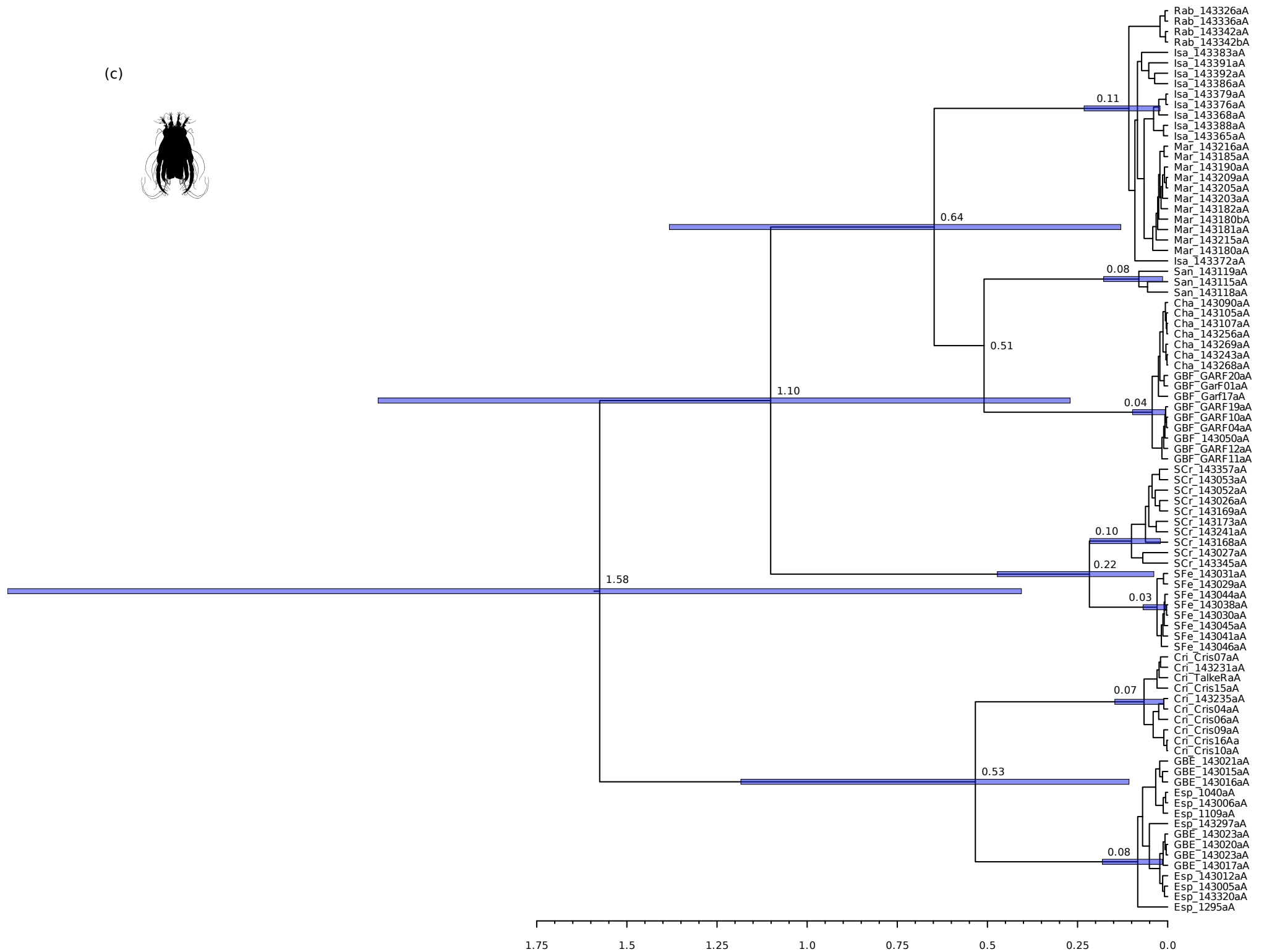

(d)

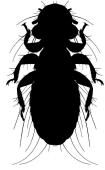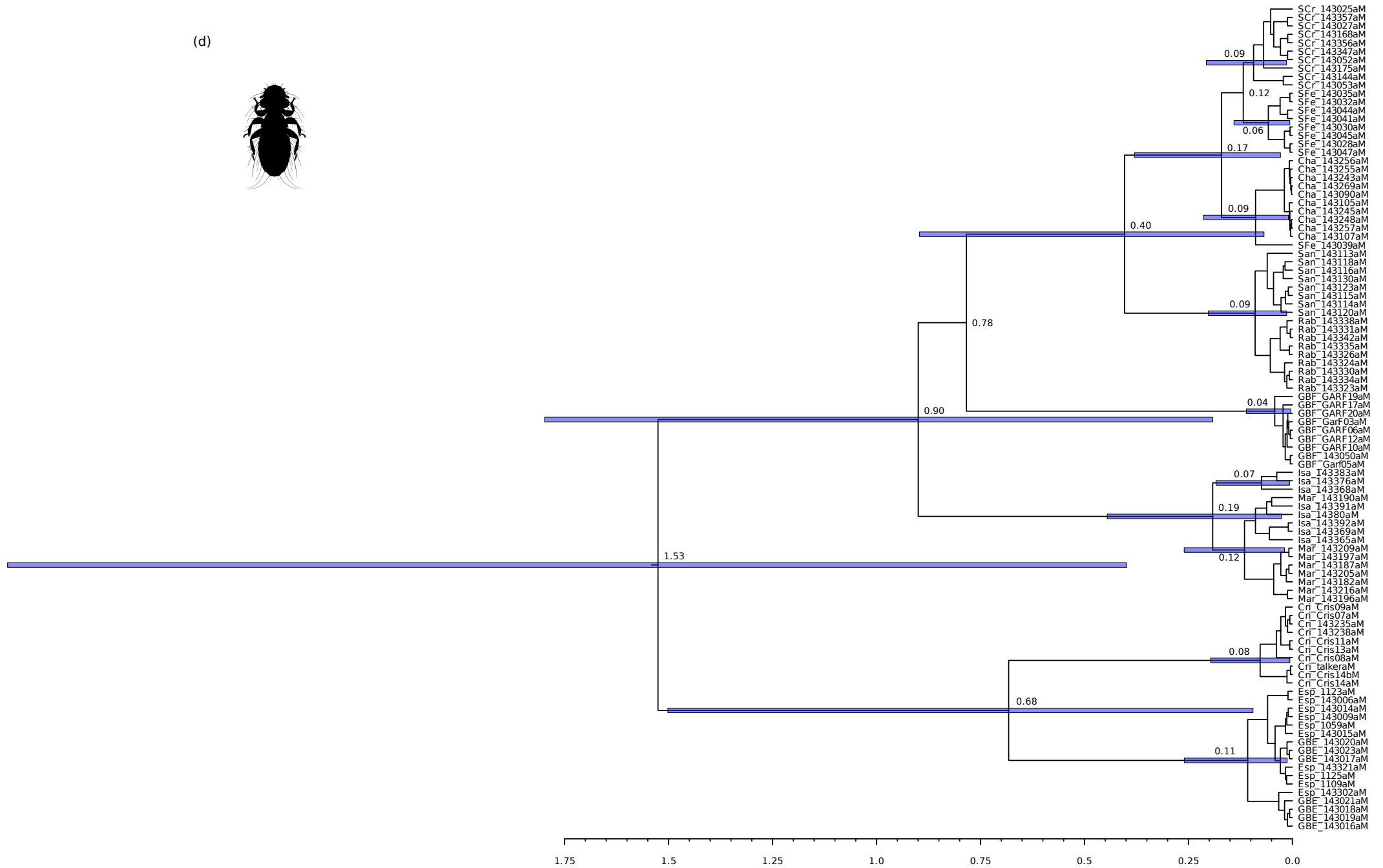

Supplement: Additional file 3 — *BEAST chronograms. Provided are multi-species chronogram (a) and chronograms for Mimus (b), Analges (c) and Myrsidea (d) datasets. Tip labels on the multi-species tree are the same as in Figure 1. Tip labels on the individual species trees are abbreviations of the island names from Figure 1 and voucher numbers from Additional file 1. Mean values of ages for major clades are provided with blue bars ranging the 95% highest posterior probability interval. [file 1471-2148-11-284-S3.PDF]

(a)

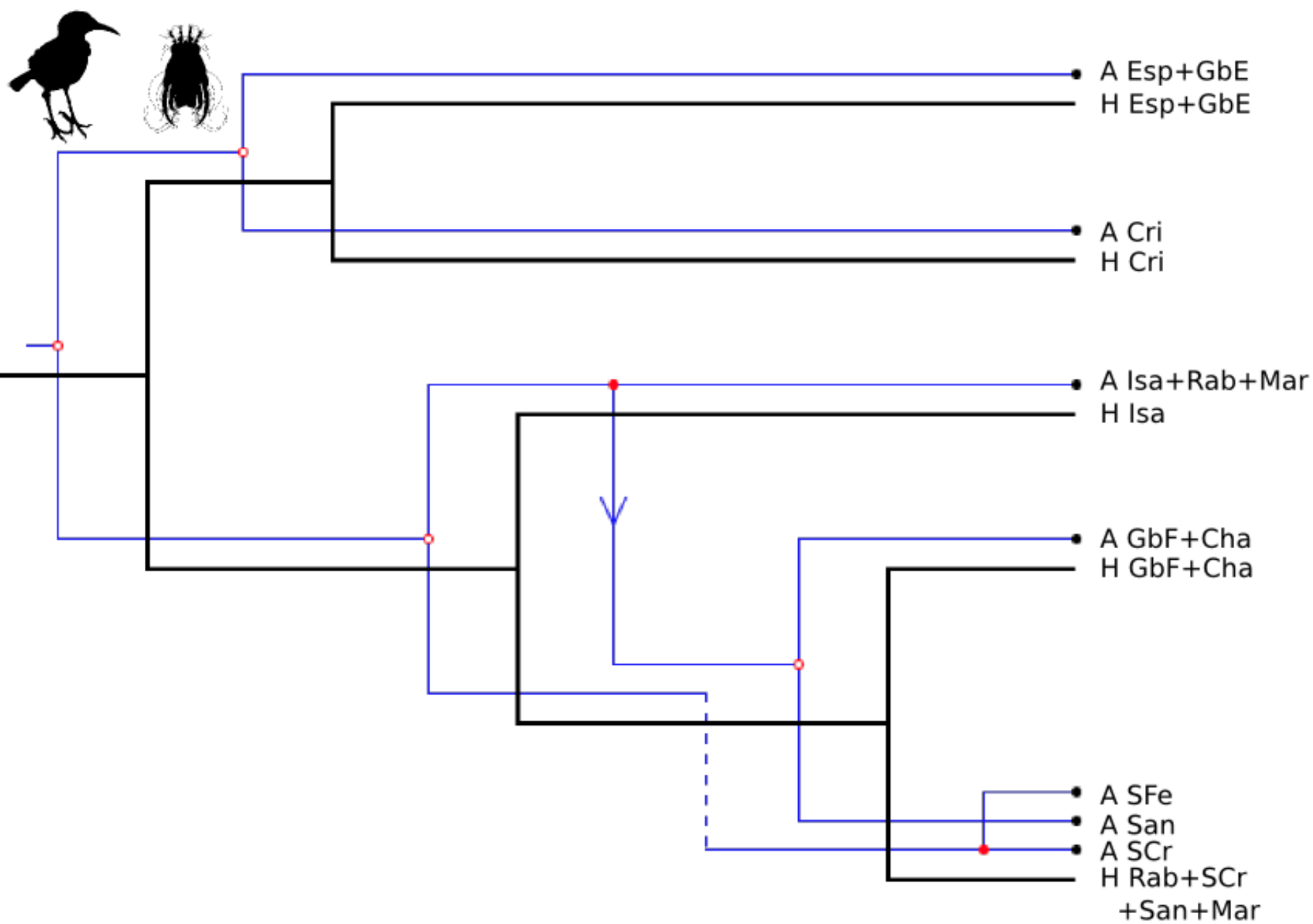

(b)

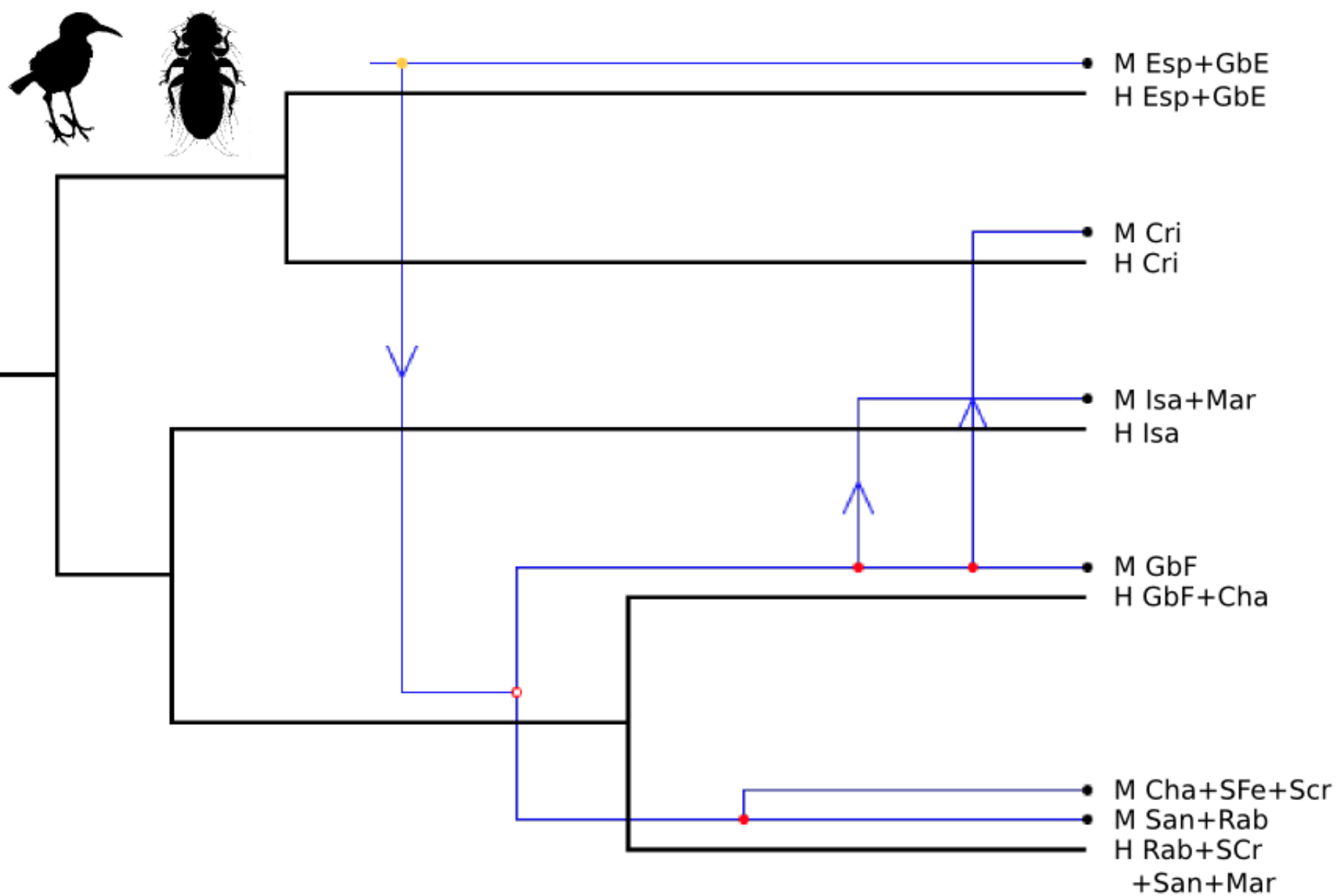

Supplement: Additional file 5 — Host-parasite phylogenies mapped with Jane software. Results for Mimus-Analges (a) and Mimus-Myrsidea (b) associations are shown. Hollow circles mark co-speciations, solid circles mark duplications. Host switches are marked by arrows and losses by dashed lines. Host trees are in black, mapped parasite histories are in blue. Yellow nodes indicate another location of equal cost exists, red nodes mark the solution with the lowest cost. Taxon labels were assigned as species and island abbreviations as shown in Figures 1 and 2. [file 1471-2148-11-284-S5.PDF]
